# A comprehensive review of physiological and molecular responses to stress of lilies (genus *Lilium*)

**DOI:** 10.1093/hr/uhaf347

**Published:** 2025-12-09

**Authors:** Yuchao Tang, Bohao Tan, Huiyu Liu, Yijie Liu, Lu Zhang, Peng Zhang, Ming Sun

**Affiliations:** State Key Laboratory of Efficient Production of Forest Resources, Beijing Key Laboratory of Ornamental Plants Germplasm Innovation and Molecular Breeding, Beijing Laboratory of Urban and Rural Ecological Environment, National Engineering Research Center for Floriculture, School of Landscape Architecture, Beijing Forestry University, Beijing 100083, China; State Key Laboratory of Efficient Production of Forest Resources, Beijing Key Laboratory of Ornamental Plants Germplasm Innovation and Molecular Breeding, Beijing Laboratory of Urban and Rural Ecological Environment, National Engineering Research Center for Floriculture, School of Landscape Architecture, Beijing Forestry University, Beijing 100083, China; State Key Laboratory of Efficient Production of Forest Resources, Beijing Key Laboratory of Ornamental Plants Germplasm Innovation and Molecular Breeding, Beijing Laboratory of Urban and Rural Ecological Environment, National Engineering Research Center for Floriculture, School of Landscape Architecture, Beijing Forestry University, Beijing 100083, China; State Key Laboratory of Efficient Production of Forest Resources, Beijing Key Laboratory of Ornamental Plants Germplasm Innovation and Molecular Breeding, Beijing Laboratory of Urban and Rural Ecological Environment, National Engineering Research Center for Floriculture, School of Landscape Architecture, Beijing Forestry University, Beijing 100083, China; State Key Laboratory of Efficient Production of Forest Resources, Beijing Key Laboratory of Ornamental Plants Germplasm Innovation and Molecular Breeding, Beijing Laboratory of Urban and Rural Ecological Environment, National Engineering Research Center for Floriculture, School of Landscape Architecture, Beijing Forestry University, Beijing 100083, China; State Key Laboratory of Efficient Production of Forest Resources, Beijing Key Laboratory of Ornamental Plants Germplasm Innovation and Molecular Breeding, Beijing Laboratory of Urban and Rural Ecological Environment, National Engineering Research Center for Floriculture, School of Landscape Architecture, Beijing Forestry University, Beijing 100083, China; State Key Laboratory of Efficient Production of Forest Resources, Beijing Key Laboratory of Ornamental Plants Germplasm Innovation and Molecular Breeding, Beijing Laboratory of Urban and Rural Ecological Environment, National Engineering Research Center for Floriculture, School of Landscape Architecture, Beijing Forestry University, Beijing 100083, China

## Abstract

Global climate change and widespread unsustainable agricultural practices increasingly impose both biotic and abiotic stresses on the production of horticultural plants. Lilies (*Lilium* spp.) are globally renowned ornamental plants, with some species also possessing medicinal, edible, and cosmetic value. However, their quality and yield are often negatively affected by various stresses. Conventional breeding methods are often inefficient due to the long juvenile phase, complex genetic background, and large genome size of lilies. While numerous emerging technologies provide opportunities for resistance breeding in lilies, their successful application relies on a thorough understanding of the resistance response mechanisms. This review systematically summarizes recent advances in lily stress resistance research, delineating the physiological and molecular response mechanisms of lilies under abiotic stresses (extreme temperature, drought, high salinity), biotic stresses (pathogens, pests), and continuous cropping obstacles. Furthermore, it discusses current challenges and limitations, and explores the potential applications of emerging technologies in improving the stress adaptability of lilies. These findings provide important insights for advancing stress resistance research and breeding stress-tolerant lily cultivars.

## Introduction

Lilies (genus *Lilium*), perennial herbaceous plants within the family Liliaceae, represent a globally important ornamental crop with additional edible, medicinal, and nutritional value [1, 2]. As one of the world’s most prominent bulbous ornamental plants, this genus currently comprises approximately 120 accepted species worldwide [3], alongside over 10 000 registered cultivars and more than 300 new cultivars registered annually [4]. Owing to their high ornamental value, lilies are widely utilized in cut flower production, potted plant cultivation, and landscape greening [1, 5, 6]. Moreover, as a genus rich in chemical diversity, lilies have long been a focus in natural product chemistry. They contain a diverse type of bioactive compounds, including phenolic acids, flavonoids, saponins, alkaloids, and polysaccharides [2, 7–11]. These constituents underpin their traditional use as food, herbal medicine, and dietary supplements in various regions. So far, over 30 *Lilium* species have been historically documented as food or medicinal resources [12]. For example, the Chinese Pharmacopoeia recognizes *Lilium lancifolium*, *Lilium pumilum*, and *Lilium brownii* var. *viridulum* as medicinal lilies with functions such as nourishing yin, moistening the lungs, calming the mind, and soothing the nerves [13]. Modern pharmacological studies have further revealed a range of physiological effects of lilies, including antioxidant, anti-inflammatory, antitumor, antifatigue, antidepressant, hypoglycemic, immunoenhancing, and neuroprotective activities [9, 14–16], highlighting their potential in disease treatment and health promotion [11]. Additionally, with the rapid growth of the cosmetics industry, lily extracts are increasingly employed as natural ingredients in skincare products for their antioxidant, whitening, antiaging, and moisturizing properties [17].

Valued for their significant and diverse applications, lilies command considerable global attention. Their widespread cultivation across multiple countries and regions generates considerable economic benefits locally. According to the International Association of Horticultural Producers (IAPH/AIPH), European countries exported lilies worth more than EUR 146.1 million in 2023, and the United States reported a production value of USD 45.3 million for cut lilies and USD 24.7 million for potted Easter lilies [18]. As the largest producer of cut lilies, China has a cultivation area exceeding 6000 hectares. In addition, the combined annual production area for edible and medicinal lilies cultivated in the open field in China surpasses 20 000 hectares [19].

However, increasing conflicts between human activities and the ecological environment, along with notable global climate changes, profoundly affect plant growth and development [20]. Throughout their life cycle—from bulb propagation and cut flower production to storage and transportation—lilies encounter various abiotic stresses (e.g. cold, heat, salinity, drought) and biotic stresses (e.g. diseases and pests) [21–23]. These stresses pose serious constraints to large-scale cultivation and routine horticultural management, underscoring the importance of research on lily stress resistance. To address these challenges, researchers have sought to elucidate the stress response mechanisms in lilies and refine the associated regulatory networks through physiological and molecular approaches, thereby establishing a foundation for stress-resistant breeding. Nevertheless, a comprehensive synthesis of advances in this field remains lacking. This review consolidates global progress in understanding lily responses to biotic and abiotic stresses, highlights key findings and existing knowledge gaps, and proposes future research directions. The integrated perspectives provided herein offer a valuable reference for advancing sustainable lily production systems.

## Responses to abiotic stress of lily

### Heat stress

**Figure 1:**
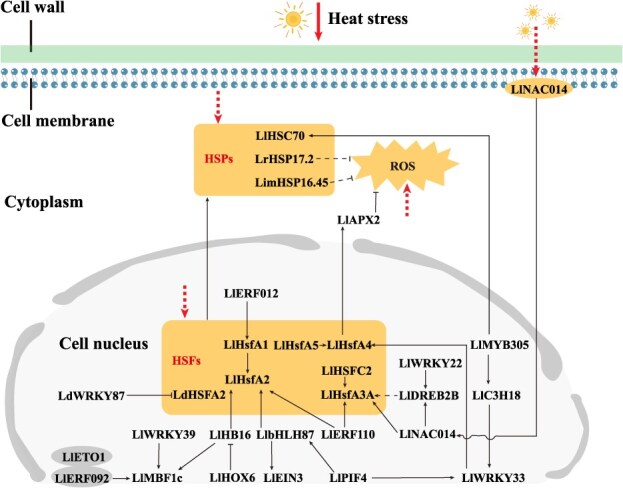
Physiological and molecular mechanisms of lily response to heat stress. Sharp arrow indicates activation, while blunt arrow indicates inhibition. Broken lines indicate indirect manipulation of a process or unknown mechanism. The thick dashed arrows represent the heat stress-induced signals.

Lilies exhibit optimal growth under cool climatic conditions. When exposed to high temperature, they often show vegetative growth retardation, developmental delays, reduced flower size, increased incidence of blind flowers, and greater susceptibility to diseases [24, 25]. The HSF–HSP module serves as a critical regulator of plant heat responses. Current research on thermotolerance in lilies remains largely centered on heat shock proteins (HSPs) and heat shock transcription factors (HSFs). Transcriptomic analysis of *Lilium longiflorum* under heat stress has revealed rapid upregulation of HSF family members and small HSPs such as HSP20 during early stress responses. Moreover, LlHsfA2 interacts with LlHsfA1 to activate downstream genes including *Hsp101*, *Hsp70*, and *Hsp25.3*, thereby positively regulating thermotolerance [25–27]. LlHSFC2 has also been shown to enhance thermotolerance through interactions with multiple HSFA family members [28] (Fig. 1). Additionally, LlHsfA4 from *L. longiflorum*, LrHSP17.2 from *Lilium regale*, and LimHSP16.45 from *Lilium davidii* var. *willmottiae* contribute to improved thermotolerance by maintaining reactive oxygen species (ROS) homeostasis [29–31]. The *HsfA3* gene from *L. longiflorum* enhances thermotolerance by promoting proline catabolism, though it concurrently increases sensitivity to salt stress [32]. Furthermore, the LlHsfA5–LlHsfA4 module antagonistically balances thermotolerance by modulating LlAPX2-mediated ROS homeostasis [33] (Fig. 1).

Although the HSF–HSP module constitutes the central regulatory mechanism for thermotolerance in lilies, its activity is further modulated by other signaling pathways and transcription factors. For instance, in *L. longiflorum*, the ethylene-responsive transcription factor LlERF110 interacts with LlHsfA2 to positively regulate thermotolerance [26, 34] (Fig. 1), and LlERF012 interacts with LlHSFA1 to enhance thermotolerance [35]. Under heat stress, LlNAC014 translocates from the cell membrane to the nucleus, where it activates the DREB2–HSFA3 module to promote thermotolerance [36, 37] (Fig. 1). The HD-Zip transcription factor LlHB16 from *L. longiflorum* enhances thermotolerance by activating *LlHSFA2* and *LlMBF1c* expression [38] (Fig. 1), while another HD-Zip member, LlHOX6, negatively regulates thermotolerance by suppressing *LlHB16* expressio [39] (Fig. 1). *LdWRKY87*, identified from *L. davidii* var. *willmottiae*, represses the expression of *LdMBF1c*, *LdHSP70* and *LdHSFA2* to reduce thermotolerance [40]. Moreover, LlERF092 interacts with LlETO1, promotes the expression of *LlMBF1c*, and enhances thermotolerance [41]. LlWRKY39, on the other hand, acts as a positive regulator by promoting *LlMBF1c* expression [42] (Fig. 1). LlMYB305 from *L. longiflorum* positively regulates thermotolerance through the MYB305–C3H18–WRKY33–LlHsfA4–LlCAT2 functional module and the LlMYB305–LlHSC70 module, both of which help maintain ROS homeostasis [43, 44] (Fig. 1). Additionally, LlbHLH87 enhances thermotolerance by activating *LlEIN3* and *LlHSFA2* while antagonizing LlSPT [45], and LlPIF4 positively regulates thermotolerance by activating *LlWRKY33* and physically interacting with LlbHLH87 [46] (Fig. 1). Other pathways such as phenylpropanoid biosynthesis, plant–pathogen interactions, phytohormone signaling, and kinase signaling have also been implicated in the thermotolerance of *L. longiflorum* [21]. Although their precise mechanisms remain unclear, these findings contribute to a broader conceptual framework for understanding the heat stress response in lilies.

### Salt stress

Soil salinization is a global issue that severely restricts land availability and crop productivity, impacting approximately one-fifth of the world’s agricultural land [47]. Salt stress impairs plant growth mainly through ion imbalance, osmotic stress, and oxidative damage, often resulting in wilting or plant death under severe conditions [48, 49]. As a result, the mechanisms underlying plant salt tolerance have attracted extensive research interest. As an important horticultural crop grown worldwide, lilies are also subject to the challenges posed by soil salinization. Most lily cultivars display limited salt tolerance, making salinity one of the constraints in commercial lily production.

**Figure 2:**
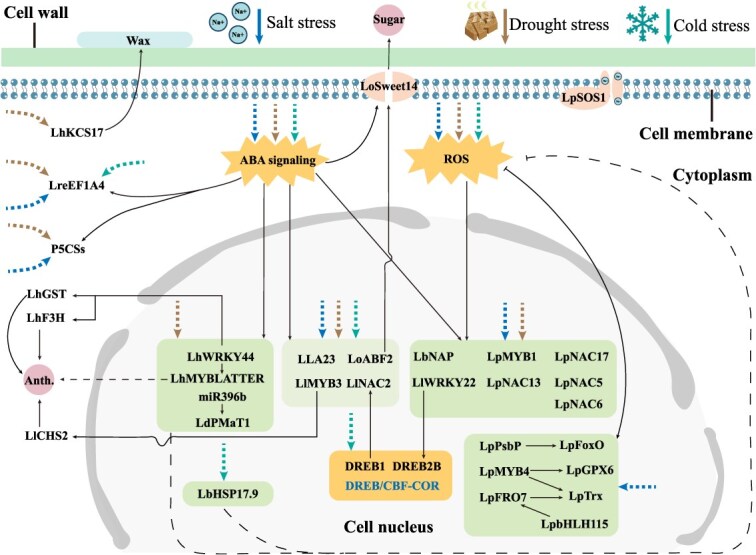
Physiological and molecular mechanisms of lily response to salt, drought and cold stress. Sharp arrow indicates activation, while blunt arrow indicates inhibition. Broken lines indicate indirect manipulation of a process or unknown mechanism. The thick dashed arrows of different colors respectively represent the corresponding stress-induced signals. Anth., anthocyanin.

Accumulating evidence underscores the critical role of abscisic acid (ABA) signaling in mediating salt tolerance in lilies [50]. For instance, the *LLA23* from *L. longiflorum* is induced by both ABA and salt stress, and its overexpression enhances salt tolerance in transgenic *Arabidopsis* [51, 52]. Similarly, transcription factors, such as *LlWRKY22* and *LlDREB2B* from *L. longiflorum* [53], *LoSWEET14* from *Lilium* oriental hybrid ‘Sorbonne’ [54] (Fig. 2), as well as *LrP5CS* and *LreEF1A4* from *L. regale*, are induced under ABA and salt stress, and their heterologous expression improves salt tolerance in transgenic tobacco or *Arabidopsis* [55, 56] (Fig. 2). In addition to ABA-related pathways, the MAPK signaling cascade also contributes to salt stress responses in lilies. For example, salt stress significantly upregulates *LlMAPK* expression in *L. lancifolium* [57], and transcriptomic analyses have revealed that multiple genes within the MAPK pathway are upregulated under salt stress in *L. pumilum* [58], supporting their involvement in salt adaptation.

Transcription factors play critical roles in plant responses to salt stress. For example, in rice, GL12 regulates the expression of the salt tolerance-associated transcription factors *NAC5* and *NCED3*, thereby positively modulating salt tolerance [59]. In *Arabidopsis*, AtWRKY9 responds to salt signals and enhances salt tolerance by regulating *AtCLCf* expression to maintain Cl^−^ distribution and homeostasis [60]. Studies have revealed that LpNAC5, LpNAC6, LpNAC13, and LpNAC17 from *L. pumilum* positively regulate salt tolerance by influencing the expression of ROS-related genes [61–65] (Fig. 2). LlNAC2 from *L. lancifolium* interacts with the AP2-type transcription factor LlDREB1 to enhance salt tolerance [66, 67] (Fig. 2). Several MYB transcription factors from *L. pumilum*, including LpMYB1 and LpMYB4, are upregulated under salt stress and improve salt tolerance by modulating ROS-related parameters [68, 69] (Fig. 2). Similarly, LlMYB3 from *L. lancifolium* responds to salt stress and ABA signaling to promote salt tolerance [70] (Fig. 2). Additionally, heat shock proteins (Hsps) and heat shock transcription factors (Hsfs) are involved in salt stress adaptation in lilies, though their regulatory roles appear multifaceted. For instance, *LfHsp70-1*, *LfHsp70-2*, *LfHsp70-3*, and *LfHsp90* from *Lilium formolongi* were rapidly induced by salt treatment, suggesting their potential role in salt stress tolerance [71]. In contrast, transgenic *Arabidopsis* expressing LlHsfA3A and LlHsfA3B from *L. longiflorum* exhibited reduced salt tolerance, likely due to altered proline catabolism and metabolism [32].

Salt stress often induces oxidative damage in plants, making ROS-related genes and enzymes a major focus in salt tolerance research [72]. Overexpression of *LpNAC17* from *L. pumilum* in tobacco significantly elevates the expression of *NtSOD*, *NtPOD*, and *NtCAT* compared to controls, thereby enhancing salt tolerance [65]. LpPsbP from *L. pumilum* mitigates salt-induced damage by scavenging ROS to protect photosystem II functionality [73] (Fig. 2). Under 100 mM NaCl stress, *LpSOS1* in *L. pumilum* is induced, and its overexpression reduces MDA content while increasing POD, SOD, and CAT activities, collectively improving salt tolerance [74] (Fig. 2). Similarly, LpMYB1 and the LpbHLH115–LpFRO7 module enhance salt tolerance by upregulating SOD, POD, and CAT activities while reducing MDA level [69, 75] (Fig. 2). In addition to antioxidative mechanisms, soluble sugars are also implicated in lily salt tolerance. For instance, LhSorABF2 in *L*. ‘Sorbonne’ responds to ABA signaling under salt stress by regulating *LhSorSWEET14*, thereby promoting soluble sugar efflux to counteract salt stress [54] (Fig. 2).

Collectively, these findings indicate that hormonal signals—particularly ABA—play crucial roles in the response and adaptation of lilies to salt stress. Transcription factor families such as NAC, MYB, Hsf, and WRKY participate in salt tolerance through both ABA-dependent and ABA-independent pathways. Moreover, ROS-related signaling and regulatory mechanisms are pivotal in mediating salt tolerance in lilies [61–65]. Despite these advances, significant knowledge gaps remain. For instance, studies in model plants such as *Arabidopsis* have demonstrated the critical roles of Ca^2+^ signaling and Na^+^/H^+^ transmembrane transport in salt tolerance [76, 77]; however, whether these pathways function similarly in lilies remains unclear. Future research should explore the potential roles and regulatory mechanisms of these pathways in modulating salt stress responses in lilies, as well as provide deeper insights into the contributions of ABA and ROS signaling in this process. Furthermore, the application of NaCl-responsive fluorescent probes such as Aza-CyBz could enable precise tracking of Na^+^ dynamics under salt stress, offering a novel approach for investigating salt tolerance mechanisms in lilies [78, 79].

### Drought stress

Drought stress primarily impairs plant growth through osmotic and subsequent oxidative stress, leading to wilting or plant death, and severely limiting the yield of economic crops [80]. In lilies, genes such as *LhSorP5CS* from ‘Sorbonne’ [81], *LlDREB1G* from *L. longiflorum* [82], *LlMYB3* from *L. lancifolium* [70], the LlNAC2–LlDREB1 module [65], and *LpNAC5* from *L. pumilum* [61] respond to ABA signaling and positively regulate drought tolerance (Fig. 2). In *Lilium distichum*, the miR396b–LdPMAT1 module enhances drought tolerance by upregulating *LdCAT3* and *LdSOD2* expression, thereby reducing ROS levels [83, 84]. Similarly, LrHSP17.2 from *L. regale* responds to drought and ABA signaling, improving drought tolerance by elevating the activities of POD, SOD, and CAT to maintain ROS homeostasis [29]. Overexpression of *LhWRKY44* in *Arabidopsis* and tobacco also enhances drought tolerance by stabilizing ROS homeostasis [85].

Although drought and salt stress responses share similarities in lilies—and some genes such as *LLA23* from *L. pumilum* enhance tolerance to both stresses when overexpressed in *Arabidopsis* [51] —other genes exhibit opposing roles. For instance, LpNAC13 and LpNAC6 positively regulate salt tolerance but negatively regulate drought tolerance in lilies [62–64] (Fig. 2). These complex and sometimes contradictory regulatory patterns highlight the need for more systematic investigation into the mechanisms underlying lily responses to different abiotic stresses. Furthermore, several studies have indicated a close relationship between drought tolerance and sugar metabolism in lilies. In *L. davidii* var. *willmottiae*, the content of soluble sugars and polysaccharides decreases under drought stress, while glucose and trehalose levels increase, with distinct sugar metabolic patterns observed across developmental stages as part of the drought response [86, 87]. Integrated transcriptomic and metabolomic analyses further reveal that drought-induced differentially expressed genes and metabolites are enriched in sugar metabolism pathways [88], underscoring the critical role of sugar metabolism in drought adaptation.

Dehydration tolerance also significantly affects the quality and vase life of cut lilies. Studies have shown that delayed water supply after harvest triggers rapid petal dehydration and senescence, reducing commercial value. Comparative analyses of petal epidermal wax layers among lily cultivars indicate a positive correlation between wax content and dehydration stress tolerance. Overexpression of *LhKCS17* in cultivar ‘Manissa’ delays cut flower senescence and enhances dehydration resistance by promoting petal wax synthesis [89, 90] (Fig. 2). Research on *L. brownii* var. *viridulum* suggests that LbNAP negatively regulates dehydration stress tolerance, although the underlying mechanisms remain unclear [91] (Fig. 2).

Exogenous application of plant growth regulators has also been shown to alter drought tolerance in lilies. For example, 24-epibrassinolide mitigates drought effects by reducing MDA content and enhancing antioxidant enzyme activity, thereby extending the postharvest longevity of cut flowers [92, 93]. Similarly, strigolactone (SL) application upregulates the expression of *LbCu/ZnSOD* and *LbMnSOD*, boosting SOD activity and improving drought tolerance, indicating a positive role for SL signaling in drought adaptation [94].

### Cold stress

Extreme low temperature is a key factor limiting plant growth and development [95]. Lilies exhibit strong cold tolerance during dormancy (bulb stage) but remain vulnerable to extreme cold during active growth and as cut flowers. The DREB1/CBF–COR/RD regulatory network plays a central role in cold adaptation in lilies. Transcriptomic analysis of *L. davidii* var. *willmottiae* under cold stress identified nine CBF family transcription factors associated with cold tolerance [96]. In *L. lancifolium*, several cold-responsive genes have been identified, including *LIDREB1/CBF*, *LIAP2/EREBP*, *LINAC2*, and *LIR2R3-MYB*. In addition, LlFAD3, Llβ-amylase, LlP5CS, and LlCLS help mitigate cold stress by regulating osmoprotectants and carbohydrate metabolism, while LlNAC2 participates in the DREB/CBF–COR pathway to enhance cold tolerance [96–98] (Fig. 2). Exogenous ABA application in *L. lancifolium* significantly activates the DREB1/CBF–COR/RD network and improves cold tolerance [99]. Furthermore, glycerol-3-phosphate acyltransferase has been shown to positively regulate cold tolerance in lilies [100]. Notably, several genes known to respond to salt, drought, or heat stress also participate in cold adaptation. These include *LlDREB1G* [82], *LlDREB1* [67], *LlMYB3* [70], and *LlA23* [52] from *L. longiflorum*, as well as *LoSWEET14* and *LbHSP17.9* from ‘Sorbonne’, which contribute to cross-tolerance against multiple abiotic stresses, including cold [54, 101].

### Other abiotic stresses

Besides heat, salt, drought, and cold stress, other extreme environmental factors can also impair lily growth or cause plant mortality. As a typical bulbous plant, most lilies exhibit limited waterlogging tolerance. Under waterlogged conditions, lilies often develop symptoms such as leaf chlorosis, wilting, stem browning, and root or bulb rot. However, studies on the mechanisms of waterlogging tolerance in lilies remain scarce. Hypoxia represents the primary challenge during waterlogging, leading to ROS accumulation and oxidative damage [102]. Research in other species, such as maize (*Zea mays*), has highlighted the importance of ROS modulation in waterlogging tolerance. For example, transcription factors ZmERF055 and ZmEREB180 enhance waterlogging tolerance by regulating ROS homeostasis [103, 104], suggesting that ROS management could be a promising research direction for lily waterlogging studies. Moreover, rapid industrialization has released heavy metals into the atmosphere and soil, causing severe environmental pollution [105]. While some plants can accumulate heavy metals, exceeding tolerance thresholds results in phytotoxicity, plant death, and contamination of plant-derived products [106]. As a multipurpose crop valued for ornamental, edible, and medicinal uses, heavy metal accumulation in lilies not only impedes plant growth but also poses potential health risks to humans. Studies indicate that edible lily bulbs can readily accumulate heavy metals, presenting challenges for safe production [107, 108]. However, research on lily tolerance mechanisms to waterlogging, heavy metals, and other abiotic stresses remains limited currently.

As summarized in Table 1, the ABA signaling pathway and ROS scavenging system play pivotal roles in mediating abiotic stress tolerance in lilies. In parallel, transcription factor families such as NAC, HSF, MYB, and WRKY significantly contribute to the regulation of these stress adaptation responses.

**Table 1:** Abiotic stress-responsive genes and their functions identified from lilies

**Resources**	**Gene**	**Main functions**	**Grade**	**References**
*L. longiflorum*	*LlHSFA1*, *LlHSFA2*	LlHSFA1 interats with LlHSFA2 to enhance downstream HSP family gene expression and improve thermotolerance.	c	[25, 26]
*L. longiflorum*	*LlHSFC2*	Interacting with HSFAs to play an active role in balancing and maintaining thermotolerance.	bc	[28]
*L. regale*	*LrHSP17.2*	Responding to ABA signaling to promote POD, SOD, and CAT synthesis, reduce ROS content, and enhance thermotolerance.	c	[29]
*L. longiflorum*	*LlHsfA3A, LlHsfA3B*	Regulating *AtbZIP11*, *AtbZIP44*, and *AtbZIP53*, and activating *AtproDH1* and *AtproDH2* to modulate proline breakdown and metabolism, enhancing thermotolerance but weakening salt tolerance.	bc	[32]
*L. longiflorum*	*LlHsfA5–LlHsfA4*	Antagonism balances thermotolerance via LlAPX2-mediated reactive oxygen homeostasis.	b	[33]
*L. longiflorum*	*LlERF110*	Activating *LlHsfA3A* expression and interaction with LlHsfA2 to enhance thermotolerance.	b	[34]
*L. longiflorum*	*LlERF012*	Activating HSF pathway to enhance thermotolerance.	bc	[35]
*L. longiflorum*	*LlNAC014*	Activating the DREB2-HSFA3 module to enhance thermotolerance.	bc	[37]
*L. longiflorum*	*LlHB16*	Activating the expression of *LlHSFA2* and *LlMBF1c* to enhance thermotolerance.	bc	[38]
*L. longiflorum*	*LlHOX6*	Inhibiting the expression of *LlHB16* to weaken thermotolerance.	bc	[39]
**L. davidii* var. *unicolor**	*LdWRKY87*	Affecting the expressions of LdMBF1c and LdHSP70 and binding the promoter of LdHSFA2 to reduce thermotolerance.	b	[40]
*L. longiflorum*	*LlERF092*	Interacting with LlETO1 promotes the expression of *LlMBF1c* to enhance thermotolerance.	a	[41]
*L. longiflorum*	*LlWRKY39*	Promoting the expression of *LlMBF1c* to enhance thermotolerance.	bc	[42]
*L. longiflorum*	*MYB305*–*C3H18*–*WRKY33*	Enhancing thermotolerance.	c	[43, 44]
*L. longiflorum*	*LlbHLH87*	Activating the expression of *LlHSFA2* and *LlEIN3* to positively regulate thermotolerance.	bc	[45]
*L. longiflorum*	*LlPIF4*	Activating LlWRKY33 and physically interacting with LlbHLH87 to positively regulate thermotolerance.	bc	[46]
*L. longiflorum*	*LlA23*	Modulating the ABA signaling pathway to enhance cold, drought, and salt tolerance.	c	[51, 52]
*L. longiflorum*	*LlWRKY22*	Modulating the ABA signaling pathway and regulating the expression of DREB family genes to enhance heat and salt tolerance.‌	c	[53]
*L.* ‘Sorbonne’	*LoABF2*–*LoSWEET14*	Response to ABA signaling to promote soluble sugar accumulation and enhance drought and salt tolerance.	c	[54]
*L. regale*	*LrP5CS1/2/3*	Regulating proline synthesis to enhance salt and drought tolerance.	c	[55]
*L. pumilum*	*L*p*NAC5*	Enhancing antioxidant enzyme activity, increasing proline and chlorophyll content, and reducing MDA content to enhance drought and salt tolerance.	c	[61]
*L. pumilum*	*LpNAC6*	Enhancing alkali tolerance, but decreased drought tolerance.	c	[62]
*L. pumilum*	*LpNAC13*	Modulating antioxidant enzymes, MDA, proline, and chlorophyll content to enhance salt tolerance while reducing drought tolerance.	c	[64]
*L. pumilum*	*LpNAC17*	Enhancing the expression of NtSOD, NtPOD, NtCAT, NtHAK1, NtPMA4, and NtSOS1 to enhance salt tolerance.	c	[65]
*L. lancifolium*	*LlNAC2-LlDREB1*	Regulating the DREB/CBF-COR and ABA signaling pathways to enhance cold, drought, and salt tolerance.	c	[67]
*L. pumilum*	*LpMYB4*	Interacting with LpGPX6 to enhance SOD and POD activity and enhance salt tolerance.	c	[68]
*L. pumilum*	*LpMYB1*	Increasing chlorophyll, proline, SOD, POD, and CAT enzyme content and reducing MDA content to enhance salt tolerance.	c	[69]
*L. lancifolium*	*LlMYB3*	Promoting anthocyanin synthesis to enhance cold resistance.	c	[70]
*L. lancifolium*	*LlMYB3*	Responding to ABA signaling to enhance cold, drought, and salt tolerance.	c	[70]
*L. pumilum*	*LpPsbP*	Scavenging ROS to protect photosystem II function and enhance salt tolerance.	c	[73]
*L. pumilum*	*LpSOS1*	Reducing MDA content and Na^+^/K^+^ ratio, and increasing POD, SOD, and CAT content to enhance salt tolerance.	c	[74]

**Table 1:** Continued

**Resources**	**Gene**	**Main functions**	**Grade**	**References**
*L. pumilum*	*LpbHLH115*–*LpFRO7*	Promoting the increase in SOD, POD, and CAT content to enhance salt tolerance.	a	[75]
*L. longiflorum*	*LlDREB1G*	Improving drought, heat and cold resistance via the ABA signaling pathway.	c	[82]
*L. distichum*	*ldi-miR396–LdPMAT1*	Reducing ROS content to enhance drought tolerance.	ac	[83, 84]
*L.* ‘Tiny Padhye’	*LhWRKY44*	Maintaining ROS homeostasis in plants to enhance drought tolerance.	bc	[85]
*L.* ‘Manissa’	*LhKCS17*	Promoting petal wax layer synthesis to enhance cut flower drought tolerance.	bc	[89]
*L. brownii* var. *viridulum*	*LbNAP*	Promoting lily petal senescence to increase sensitivity to dehydration stress.	bc	[91]
*L.* ‘Manissa’	*LbHSP17.9*	Regulating MDA content to enhance cold tolerance in cut flowers.	bc	[101]

The grade of a: stable transformation of lily for functional validation. The grade of b: transient transformation of lily for functional validation. The grade of c: stable transformation of other plant species for functional validation.

## Responses to biotic stress of lily

### Gray mold

As one of the most devastating plant diseases, gray mold severely impacts crop yield, quality, economic value, and ecosystem health [109, 110]. In lilies, gray mold is predominantly caused by *Botrytis elliptica* and *Botrytis cinerea* [111]. Both pathogens infect leaves and flower buds, leading to symptoms such as leaf scorch and abnormal bud abscission. Additionally, *Botrytis* infections during storage and transport can cause bulb rot [112], significantly compromising lily production and commercial value.

**Figure 3:**
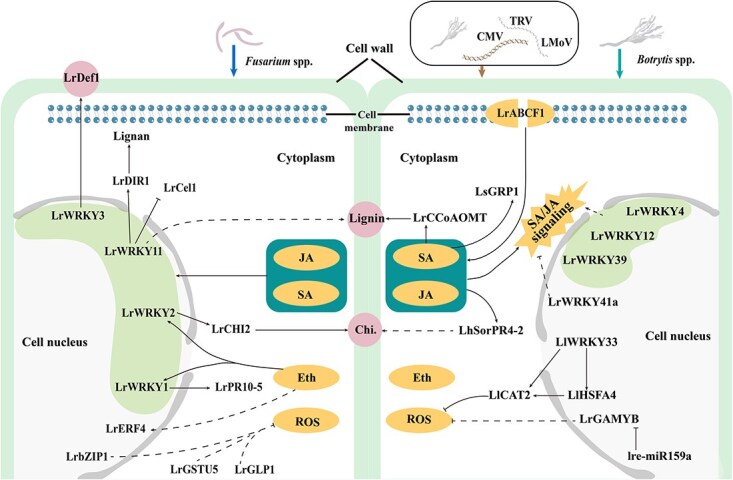
Physiological and molecular mechanisms of lily response to biotic stress. The thick arrows of different colors respectively represent the corresponding stress-induced signals. Sharp arrow indicates activation, while blunt arrow indicates inhibition. Broken lines indicate indirect manipulation of a process or unknown mechanism. Chi., Chitinase.

Gene expression analysis of *L. longiflorum* under *B. elliptica* infection showed that the expression levels of *LlWRKY3/4/5/10/12* were upregulated, indicating their involvement in the defense response [113]. Furthermore, the LlWRKY33–LlHSFA4–LlCAT2 regulatory module in *L. longiflorum* confers resistance to *B. elliptica* by reducing cell death and enhancing ROS scavenging [114] (Fig. 3). Integrated transcriptomic and functional studies in *L. regale* have identified three WRKY transcription factors—LrWRKY4, LrWRKY12, and LrWRKY39—that positively regulate resistance to *B. elliptica* by mediating salicylic acid (SA) and jasmonic acid (JA) signaling pathways, along with a negative regulator, LrWRKY41a [115, 116] (Fig. 3). These findings underscore the critical role of WRKY transcription factors in orchestrating lily defense against *B. elliptica* infection.

Recent studies have demonstrated that secondary metabolism and plant hormone signaling also contribute to lily resistance against *B. elliptica*. Functional characterization revealed that the caffeoyl-CoA *O*-methyltransferase gene *LrCCoAOMT* from *L. regale* enhances resistance to *B. elliptica* by regulating lignin biosynthesis and modulating SA signaling pathways [117] (Fig. 3). Similarly, full-length transcriptome sequencing of ‘Sorbonne’ under *B. elliptica* stress identified a pathogenesis-related gene *LhSorPR4-2*, whose expression is induced by plant hormones such as JA, SA, and ethylene. This gene enhances chitinase activity, thereby positively regulating plant resistance to *B. elliptica* [118] (Fig. 3). Moreover, integrated transcriptomic and metabolomic analyses revealed that the level of resistance to *B. elliptica* in lilies is significantly correlated with phenylpropanoid content and the transcriptional activity of genes in the phenylpropanoid biosynthesis pathway [119]. Exogenous application of melatonin has also been shown to enhance gray mold resistance in *L*. ‘Sorbonne’ and *L*. ‘Fate morgana’ [120]. A glycine-rich protein 1 gene (*LsGRP1*) mediates SA signaling and confers systemic resistance against *B. elliptica*. Notably, LsGRP1 exhibits dual antimicrobial functions by not only activating plant innate immunity but also inducing programmed cell death in *B. elliptica* [121].

In addition, miRNAs have been implicated in regulating lily resistance to *B. elliptica*. Specifically, lre-miR159a, identified in *L. regale*, positively regulates resistance by suppressing the expression of its target gene *LrGAMYB* while concurrently activating defense responses [122, 123] (Fig. 3).

The aforementioned studies on lily response to *B. elliptica* infection have primarily focused on gene identification and functional characterization, providing valuable candidate genes for resistance breeding. However, these findings have not yet substantially alleviated the production losses caused by gray mold. Notably, emerging research demonstrates that beneficial microorganisms—primarily *Trichoderma* and *Bacillus* spp.—can effectively suppress *B. elliptica* development in lilies [124, 125]. Although the precise mechanisms of these microorganisms remain incompletely characterized, growing evidence suggests they mitigate *B. elliptica* damage through multiple pathways: suppressing conidial germination and hyphal extension, inducing host defense responses, and promoting plant growth. These discoveries provide valuable insights for developing biocontrol strategies against gray mold in commercial lily cultivation.

### *Fusarium* wilt

*Fusarium* wilt primarily affects the basal stems and bulbs of lilies, causing brown necrosis or rot at the bulb base, which often leads to scale detachment, stunted growth, and eventual wilting. Using suppression subtractive hybridization, a cDNA library constructed from *Fusarium oxysporum*-infected *L. regale* roots revealed differential expression of defense-related enzyme genes, including chitinase (*PR3*), *PR10*, glutathione S-transferase (*GST*), cytochrome P450 (*CYP*), catalase (*CAT*), and peroxidases [126]. Further studies have shown that LrERF4 enhances disease resistance by promoting phenylpropanoid biosynthesis, underscoring the importance of phenylpropanoid metabolism in the defense of *L. regale* against *F. oxysporum* [127] (Fig. 3). In addition, LrWRKY1 activates the expression of the resistance gene *LrPR10-5*, while LrWRKY2 positively regulates defense by activating *LrCHI2* expression [128, 129] (Fig. 3). LrWRKY11 interacts with SA/JA signaling pathways to induce LrDIR1-mediated lignin accumulation [130], and LrWRKY3 modulates defense responses by activating SA/JA signaling and regulating *LrDef1* expression [131], collectively enhancing resistance to *F. oxysporum* (Fig. 3). The *LrbZIP1* gene isolated from *L. regale* enhances resistance to *F. oxysporum* by increasing antioxidant enzyme activity and upregulating the transcription of multiple resistance-related genes [132] (Fig. 3). *LrGSTU5* from *L. regale* responds to ABA and ethylene signaling to strengthen resistance against this pathogen [133] (Fig. 3). In contrast, the expression of *LrCel1* is suppressed by *F. oxysporum* and SA, and its overexpression in tobacco negatively regulates resistance. It is noteworthy that LrWRKY11, a positive regulator of *Fusarium* wilt resistance in *L. regale*, inhibits *LrCel1* expression [134] (Fig. 3). Additionally, LrGLP1 in *L. regale* enhances resistance to *Fusarium* wilt by inducing *MnSOD*, *Cu/ZnSOD*, and *PR1*, thereby elevating superoxide dismutase (SOD) and oxalate oxidase (OXO) activities and improving ROS scavenging capacity [135] (Fig. 3).

Current management of *Fusarium* wilt primarily relies on chemical methods, including disinfection, soil treatment, and fungicide root drenching. Studies demonstrate that exogenous aconitic acid reduces disease severity in *L. davidii* var. *willmottiae* and functions as an allelopathic inhibitor in *Fusarium* wilt control [136]. In addition, antagonistic microorganisms such as nonpathogenic *F. oxysporum* [137] and other biocontrol bacteria can suppress plant diseases through mechanisms such as direct antagonism, nutrient and spatial competition, hyperparasitism, and induced systemic resistance.

### Viral diseases

More than 20 viral diseases have been reported in lilies [138, 139]. The most prevalent viruses include Lily symptomless virus (LSV), Cucumber mosaic virus (CMV), and Lily mottle virus (LMoV), which infect leaves, stems, flowers, buds, and bulbs, resulting in symptoms such as stunting, growth arrest, and leaf malformation. Effective viral disease management relies on accurate detection methods [140]. Advances in molecular technologies have enhanced the identification of viral species, with previous studies characterizing 12 complete and 6 near-complete viral genomes and analyzing their recombination and phylogenetic relationships to improve control strategies for lily viral diseases [138]. A transcriptomic study of *L. regale* under CMV stress identified an ABC transporter gene, *LrABCF1*, which positively regulates resistance to both viral and fungal pathogens [141] (Fig. 3). Furthermore, virus–fungus interactions and virus-induced alterations in lily rhizospheric metabolites and microbial communities have been shown to influence soil carbon and nitrogen dynamics. The implications of these microbial community changes for lily growth and nutritional quality merit further investigation.

Table 2 summarizes numerous genes involved in biotic stress regulation that have been identified in *Lilium* species. The available data indicate that hormonal signaling pathways mediated by SA and JA, ROS signaling, and the phenylpropanoid biosynthesis pathway function as central regulatory hubs in the biotic stress responses of lilies. In addition, transcription factor families, particularly WRKY, are extensively involved in orchestrating these defense mechanisms.

**Table 2:** Biotic stress-responsive genes and their functions identified from lilies

**Resources**	**Gene**	**Functions**	**Grade**	**Reference**
*L. longiflorum*	*LlWRKY33–LlHSFA4–LlCAT2*	Reducing cell death and ROS accumulation to enhance resistance against *B. cinerea*.	bc	[114]
*L. regale*	*LrWRKY4/12*	Enhancing resistance to *B. cinerea* through SA and JA signaling pathways.	c	[115]
*L. regale*	*LrWRKY39*	Enhancing resistance to *B. cinerea* through SA and JA signaling.	bc	[116]
*L. regale*	*LrWRKY41a*	Weakening resistance to *B. cinerea* through SA and JA signaling.	bc	[116]
*L. regale*	*LrCCoAOMT*	Increasing lignin deposition to enhance resistance against *B. cinerea*.	bc	[117]
*L.* ‘Sorbonne’	*LhSorPR4-2*	Response to JA, SA, and ET induction elevates chitinase activity and enhances resistance against *B. elliptica*.	a	[118]
*L. regale*	*Lre-miR159a*	Inhibiting *LrGAMYB* gene expression and activating defense responses to enhance resistance against *B. elliptica*.	c	[123]
*L. regale*	*LrERF4*	Promoting phenylpropanoid biosynthesis to enhance lily resistance against *F. oxysporum*.	b	[127]
*L. regale*	*LrWRKY1, LrPR10-5*	LrWRKY1 activates *LrPR10-5* expression to enhance resistance against *F. oxysporum.*	ac	[128]
*L. regale*	*LrWRKY2, LrCHI2*	LrWRKY2 activates *LrCHI2* expression to enhance resistance against *F. oxysporum.*	bc	[129]
*L. regale*	*LrWRKY11, LrDIR1*	LrWRKY11 activates *LrDIR1* expression through SA/JA signaling pathways to accumulate lignans and enhance resistance against *F. oxysporum.*	bc	[130]
*L. regale*	*LrWRKY3*	Activating the expression of disease resistance genes like *LrDef1* via SA/JA signaling to enhance resistance against *F. oxysporum.*	bc	[131]
*L. regale*	*LrbZIP1*	Enhancing antioxidant enzyme activity and partial resistance-related gene expression to strengthen resistance against *F. oxysporum.*	c	[132]
*L. regale*	*LrGSTU5*	Positively regulates disease resistance genes and antioxidant enzyme systems to enhance resistance against *F. oxysporum*.	c	[133]
*L. regale*	*LrCel1*	Negatively regulates resistance against *F. oxysporum*.	c	[134]
*L. regale*	*LrGLP1*	Enhancing ROS scavenging capacity to improve the resistance to *F. oxysporum.*	c	[135]
*L. regale*	*LrABCF1*	Positively regulating viral and fungal resistance.	c	[141]

The grade of a: stable transformation of lily for functional validation. The grade of b: transient transformation of lily for functional validation. The grade of c: stable transformation of other plant species for functional validation.

### Pests

Aphids represent one of the primary insect pests affecting lilies, with the most damaging species being the polyphagous *Aphis gossypii* (cotton aphid) and *Myzus persicae* (green peach aphid). Aphid infestations impair lily growth, reduce yield and quality, and facilitate the transmission of viruses such as LSV and LMoV [142, 143]. However, research on lily resistance to aphids remains limited, with only small-scale evaluations of aphid resistance and transcriptomic analyses currently available [23, 144]. Plants have evolved diverse defense mechanisms against aphids, including physical barriers such as lignin [145, 146] and callose deposition [147], as well as secondary metabolites that directly repel or kill aphids [148]. Signaling pathways involving SA and JA play central roles in plant–aphid interactions. For instance, *Sitobion miscanthi* (grain aphid) secretes SmCSP4, which interacts with *Triticum aestivum* TaWRKY76 to suppress SA-mediated immunity [149], whereas JA signaling is critical for resistance to *Rhopalosiphum maidis* (corn leaf aphid) in *Z. mays* [150]. Recent studies also highlight the role of methyl salicylate (MeSA), a key airborne defense signal that is perceived by plant SABP2 proteins, converted to SA, and regulates aphid resistance via the NAC2–SAMT1 module [151, 152]. These findings provide valuable insights for advancing research on aphid resistance in lilies.

Other pests, including thrips (Thripidae), bulb mites (Acaridae), *Bradysia odoriphaga*, *Pratylenchus penetrans* (root lesion nematode), *Bemisia tabaci* (whitefly), and click beetles (Elateridae), also adversely affect lily growth and development [153, 154]. Nevertheless, studies on lily defense responses against these pests are still limited.

### Continuous cropping obstacles

**Figure 4:**
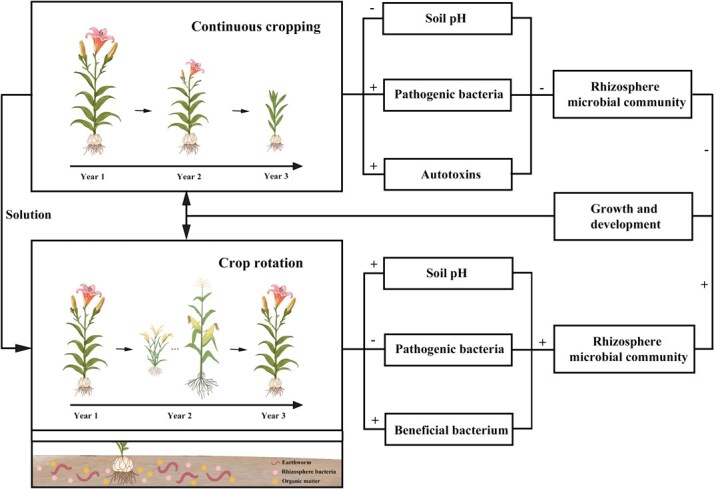
Potential formation mechanisms and mitigation strategies for lily continuous cropping obstacle. ‘+’ represents promotion, while ‘−’ indicates inhibition.

Although cut lily production increasingly relies on protected cultivation and soilless substrate systems, the production of medicinal and edible lilies often employs continuous cropping due to constraints related to Geographical Indication product requirements. This practice leads to continuous cropping syndrome, characterized by root rot, replant disease, soilborne pathogen accumulation, and yield loss, ultimately threatening both crop productivity and soil sustainability [155]. Continuous cropping significantly alters the physicochemical properties and microbial composition of rhizosphere soil, thereby reducing lily quality and yield [156, 157]. For example, continuous cropping of *L. lancifolium* decreases soil pH and promotes the enrichment of pathogenic genera such as *Pseudomonas*, *Streptomyces*, and *Fusarium* in the rhizosphere, resulting in root rot [156, 158] (Fig. 4). Similarly, continuous cropping of *L. davidii* var. *willmottiae* facilitates the accumulation of *Fusarium* spp., thereby triggering disease outbreaks [159].

The accumulation of allelopathic autotoxins represents another critical factor contributing to continuous cropping obstacles. Phthalic acid, a major autotoxin secreted by the roots of *L. davidii* var. *willmottiae*, inhibits root elongation, reduces root activity, impairs leaf photosynthesis, and suppresses overall plant growth when it accumulates in the rhizosphere [160]. Another autotoxin, 2,4-di-*tert*-butylphenol, promotes the proliferation of pathogenic *Fusarium* spp. while reducing populations of beneficial microorganisms, thereby exacerbating disease incidence in lilies [161]. Additionally, 4-vinyl guaiacol, derived from phenolic degradation in *L. davidii* var. *willmottiae*, enhances the pathogenicity of *F. oxysporum* strain B4, further reducing crop yields [162].

Crop rotation currently serves as the primary mitigation strategy for continuous cropping obstacles. Studies demonstrate that rotating lilies with rice elevates soil pH, activates JA biosynthesis, promotes the proliferation of beneficial *Aspergillus* spp., suppresses fungal pathogens such as *Fusarium* and *Botrytis*, and enhances the synthesis of medicinal alkaloids in bulbs, ultimately improving yield [158, 163, 164]. Similarly, intercropping *L. davidii* var. *willmottiae* with maize enriches beneficial *Sphingomonas* spp. while suppressing harmful *Fusarium* populations [159] (Fig. 4). Earthworm activity has also been shown to alleviate continuous cropping obstacles in *L. lancifolium* by increasing soil organic matter (OM), available nitrogen (AN), phosphorus (AP), and potassium (AK), optimizing bacterial community structure and diversity, and elevating the abundance of beneficial taxa such as Rhizobiales, Myxococcales, Streptomycetales, and Pseudomonadales [165] (Fig. 4).

In summary, continuous cropping obstacles pose significant challenges to sustainable lily production. While crop rotation can partially mitigate these issues, its efficiency is often limited by land availability and logistical constraints. Recent breakthroughs in other cropping systems offer promising alternatives. For example, *Bacillus cereus* strain WL08 degrades the autotoxin butylated hydroxytoluene, alleviating continuous cropping obstacles in *Pinellia ternata* [166], and biochar application reduces fungal pathogen loads while activating soil polyphenol oxidase activity in tobacco systems [167]. Future research on lily continuous cropping obstacles should prioritize microbial regulation, green exogenous amendments, and in-depth analyses of rhizosphere microbial communities and autotoxin dynamics. Optimizing cultivation practices and establishing sustainable field management systems will be essential for achieving high-quality and high-yield lily production.

### Critical findings and knowledge gaps

Biotic and abiotic stresses significantly impact plant growth and development [168]. To cope with diverse environmental pressures, plants have evolved complex physiological and molecular regulatory networks [169]. Deciphering these mechanisms can enhance agricultural management efficiency and facilitate the breeding of stress-resistant, high-yield varieties to address challenges posed by environmental changes [170]. Existing research indicates that the ROS system plays a central regulatory role in *Lilium* responses to both biotic and abiotic stresses. Phytohormone signaling is also critical: the ABA pathway predominantly regulates abiotic stress responses, whereas SA and JA signaling pathways are more prominent in biotic stress resistance. Additionally, multiple transcription factor families participate in lily stress adaptation. The functions of HSP and HSF families in heat stress have been systematically elucidated [28, 29, 32, 71, 101], and the regulatory roles of NAC members in salt and drought stress are increasingly understood [61, 62, 64, 65, 67, 70]. WRKY transcription factors are pivotal in biotic stress responses [113–116, 128–131], while other families such as MYB, bZIP, and bHLH are also implicated in stress regulation [68, 70, 75, 132]. Concurrently, the involvement of specific microRNAs in lily stress resistance has been preliminarily documented [83, 84, 123]. Notably, recent studies highlight the importance of secondary metabolism—particularly phenylpropanoid metabolism and related pathways—in lily stress adaptation [89, 127, 129, 130], expanding the scope of stress resistance research. In summary, substantial progress has been made in understanding lily responses to salt, drought, heat, cold, and pathogens such as *Fusarium*, *Botrytis*, and viruses.

By contrast, research on waterlogging, heavy metal stress, and insect pests remains limited, despite their significant threats to lily production. Moreover, studies on dehydration and senescence in cut lilies—one of the world’s most important fresh-cut flowers—are notably insufficient. Research on tulips, which share physiological traits with lilies, may offer valuable insights. For instance, transcription factors such as TgNAP, TgWRKY75, and TgWRKY44 in tulips promote petal senescence by mediating SA and ABA signaling pathways [171–173]. Understanding these genetic functions provides critical insights for developing antisenescence strategies in cut lilies. Similarly, recent studies show that LoZAT12 accelerates senescence in cut lilies by activating SA and ABA biosynthesis [174]. Furthermore, emerging evidence indicates that citrulline can reduce cadmium (Cd) uptake by enhancing the lignin barrier, while zinc oxide nanoparticles synergistically decrease Cd accumulation in *Fritillaria cirrhosa* via coordinated activation of antioxidant enzymes and metabolic pathways [175, 176]. These findings suggest promising approaches for mitigating heavy metal accumulation in edible and medicinal lilies. On the other hand, substantial research has been conducted on lily responses to heat, salt, and pathogen stresses. These advances provide valuable references for related studies in other Liliaceae species with similar life forms. For example, pathogens such as *Botrytis* spp. and *Fusarium* spp. also cause bulb rot in tulips, with *Botrytis tulipae* capable of inducing complete bulb decay [177].

Furthermore, current research predominantly focuses on limited species. A systematic evaluation of stress tolerance across *Lilium* species is lacking, which may hinder the discovery and utilization of elite stress-tolerant germplasm and obstruct breeding progress and mechanistic studies. Additionally, most studies emphasize physiological responses and gene expression profiling. Although functional analyses of key genes have been initiated, incomplete population genomic data, robust genetic transformation, and gene editing systems in lilies restricts functional validation in native plants. The impacts of these genes on growth and developmental traits also remain unclear, further limiting their practical application. Moreover, current research primarily examines single-stress responses, whereas lilies in natural environments often face combined stresses—a critical yet underexplored area. Notably, several studies demonstrate that single transcriptional regulators can modulate multiple stress responses. For instance, LrWRKY16 in *L. regale* activates *LrHSP17.2* expression to maintain cellular homeostasis and enhance ROS scavenging, thereby improving tolerance to both high temperature and drought [178]. Another recent study revealed that LlWOX11 in *L. lancifolium* regulates *LlLRP1* in response to salt stress and promotes bulbil formation, offering insights into the coordinated enhancement of stress resistance and growth in lilies [179].

## Future perspectives

### Systematic germplasm resource investigation and evaluation of lilies

As economically important horticultural plants, lilies possess remarkable germplasm diversity that represents a strategic genetic reservoir for cultivar improvement. However, a substantial number of species remain underutilized [4, 180]. Meanwhile, climate change and expanding human activities have pushed some lily resources to the brink of extinction, underscoring the urgent need to enhance resource investigation, evaluation, and conservation [181]. Therefore, establishing a standardized and practical evaluation system for systematic germplasm assessment is essential. Such efforts should encompass not only traditional phenotypic data [182], but also integrate environmental parameters such as geography, climate, and soil properties. Furthermore, developing a multidimensional resistance scoring model—by integrating environmental, phenotypic, and physiological datasets through stress simulation and quantitative screening—will enable systematic evaluation of lily tolerance to both individual and combined stresses. This approach will provide a scientific basis for utilizing wild resources and selecting breeding materials. However, given the vast diversity within the genus *Lilium* and its wide distribution across Asia, Europe, and North America, this systematic effort will likely require collaboration across institutions and national borders [4].

### Leveraging novel technologies for stress resistance improvement in lily production management

In current production systems, cut flower lilies are predominantly cultivated under protected environments, which provide optimal growth conditions. However, lilies grown for landscaping, home gardening, bulb propagation, and edible or medicinal purposes often rely on open-field cultivation. The complex field environments—subject to diverse biotic and abiotic stresses—significantly compromise lily quality and yield. Conventional agrochemicals not only risk inducing pathogen and pest resistance but also pose environmental and human health hazards. Emerging nanomaterial-based solutions show promise in plant disease and pest management [183], offering novel strategies for lily cultivation. Concurrently, microbial biocontrol agents are gaining prominence in crop production. These beneficial microorganisms enhance plant resistance to both biotic and abiotic stresses while improving crop quality and yield [184]. Certain microbes also ameliorate rhizosphere soil conditions by reducing harmful substances and pathogen loads, providing an effective biological approach to mitigate continuous cropping obstacles in lilies [185]. Therefore, applying these emerging technologies to lily production management to enhance stress tolerance warrants in-depth investigation.

### Accelerating the discovery and application of resistance genes in lilies by leveraging modern biotechnology

Modern biotechnology tools can significantly enhance the precision of stress-resistance gene identification in crops and improve the efficiency of breeding resistant cultivars. Although various biotechnological approaches—including molecular markers, transgenic techniques, gene editing, and integrated multiomics analyses—have been explored in lilies, their application in breeding remains limited [1, 186]. This gap is largely attributable to challenges in genomic research caused by the large and complex genome of lilies, as well as difficulties in applying transgenic and gene editing methods across their diverse germplasm. Notably, the recent released three giant genomes of *Lilium sargentiae*, *L. davidii* var. *unicolor*, and *L. regale* have provided valuable genetic resources for lily improvement [187–189]. In addition, CRISPR/Cas9-mediated gene editing has been successfully implemented to prolong the flower longevity of lilies [190]. While these achievements represent groundbreaking progress, a considerable gap remains compared to other ornamental species such as roses and chrysanthemums. Future efforts should focus on precise and rapid identification of resistance-related genes through advanced genome sequencing of diverse *Lilium* species, construction of multidimensional omics datasets, and development of lily-optimized haploid induction and gene editing systems.

### AI-assisted enhancement of stress resistance in lilies

The rapid advancement of artificial intelligence (AI) is reshaping paradigms in plant genetic improvement. AI-enhanced technologies, both individually and in integration, can effectively accelerate lily breeding processes. The powerful phenotyping capabilities of AI can substantially improve the efficiency of germplasm resource evaluation [191]. Automated machine vision systems enable disease and pest diagnosis, prediction of abiotic stress severity, and assistance in large-scale weed management. For example, Biabi *et al.* developed machine vision systems based on machine learning that accurately assess water deficit levels in lily plants and identify *B. elliptica* infections [192, 193], providing technical support for precision irrigation and disease control. Given the large and complex genome of *Lilium* species, genomic research faces considerable challenges. However, DNA language models trained on high-quality genome data from model plants can be transferred cross-species to assist in lily genome assembly and annotation. For instance, the PlantCaduceus model developed by Zhai *et al.* enables accurate cross-species gene annotation from the eudicot *Arabidopsis thaliana* to the monocot *Z. mays* [194]. Currently, functional gene studies in lilies remain relatively limited. Research relying solely on molecular experiments is not only costly and inefficient but also constrained by inherent technical barriers in genetic transformation. Transfer learning AI strategies can leverage knowledge from functionally validated genes and regulatory networks in other plants to predict gene functions in lilies, significantly improving the screening efficiency of key genes. This approach maximizes existing data resources while avoiding redundant studies. For example, by transferring knowledge from *Arabidopsis* to cultivated tomato, Moore *et al.* significantly improved the prediction accuracy of secondary/specialized metabolism-related genes in tomato [195]. Furthermore, AI technologies can evaluate the efficiency and specificity of CRISPR/Cas systems, base editors, and prime editors, and assist in analyzing CRISPR screening results, thereby accelerating the development of lily-specific gene editing tools.

Additionally, AI’s robust capacity for large-scale data analysis and multidimensional data integration can significantly enhance breeding efficiency through multimodal data fusion and intelligent modeling. However, applying this strategy to lily genetic improvement still requires considerable effort. Although databases such as Lily-db have been developed to catalog lily morphology, molecular markers, gene expression, and transcription factors, their content remains limited [196]. Future work should prioritize the construction of comprehensive multiomics databases—encompassing genomic, phenomic, transcriptomic, proteomic, metabolomic, and environic data—and apply transfer learning to identify conserved stress-resistance pathways from model plants. Generative adversarial networks could simulate gene expression patterns under diverse stress conditions to optimize stress-resistance gene combinations, thereby providing targeted strategies for breeding new lily varieties with enhanced resistance [197]. Nevertheless, this systematic effort will require collaborative contributions from interdisciplinary and interinstitutional researchers.
